# Increased Incidence of Invasive Fusariosis with Cutaneous Portal of Entry, Brazil

**DOI:** 10.3201/eid1910.120847

**Published:** 2013-10

**Authors:** Marcio Nucci, Andrea G. Varon, Marcia Garnica, Tiyomi Akiti, Gloria Barreiros, Beatriz Moritz Trope, Simone A. Nouér

**Affiliations:** University Hospital, Universidade Federal do Rio de Janeiro, Rio de Janeiro, Brazil

**Keywords:** Fusarium, invasive fusariosis, portal of entry, skin, Brazil, fungi, cutaneous, immunocompromised, immunocompetent, Fusarium solani, Fusarium oxysporum, environmental, community, transmission, spread

## Abstract

Invasive fusariosis (IF) is an infection with *Fusarium* spp. fungi that primarily affects patients with hematologic malignancies and hematopoietic cell transplant recipients. A cutaneous portal of entry is occasionally reported. We reviewed all cases of IF in Brazil during 2000–2010, divided into 2 periods: 2000–2005 (period 1) and 2006–2010 (period 2). We calculated incidence rates of IF and of superficial infections with *Fusarium* spp. fungi identified in patients at a dermatology outpatient unit. IF incidence for periods 1 and 2 was 0.86 cases versus 10.23 cases per 1,000 admissions (p<0.001), respectively; superficial fusarial infection incidence was 7.23 versus 16.26 positive cultures per 1,000 superficial cultures (p<0.001), respectively. Of 21 cases of IF, 14 showed a primary cutaneous portal of entry. Further studies are needed to identify reservoirs of these fungi in the community and to implement preventive measures for patients at risk.

## Abstract 2

Most cases of infection with *Fusarium* spp. fungi involved primary skin lesions.

Invasive fusariosis (IF) is a mycosis caused by infection with *Fusarium* spp. fungi that affects primarily patients with hematologic malignancies and hematopoietic cell transplant (HCT) recipients (*1*,*2*). In these severely immunosuppressed patients, IF is typically disseminated and involves pneumonia, metastatic skin lesions, and positive blood cultures (*3*). The usual portal of entry is the airways, and IF is thought to be acquired by the inhalation of aerosols of fusarial conidia. However, the skin at sites of tissue breakdown may be a portal of entry (*4*). In a review of 232 published cases of IF in immunosuppressed patients, primary skin lesions represented the likely portal of entry in 16 (11%) of 147 patients with disseminated disease (*5*).

In 2007, we observed an increase in the incidence of IF in our hospital in Brazil: 5 cases of disseminated IF and 2 cases of locally invasive disease were diagnosed in a 7-month period. In addition to this apparent increase in incidence, we observed that all patients had a primary skin lesion on the lower limbs. All case-patients were housed in 4 rooms of the hematology unit. Because of the increasing incidence and the cutaneous primary lesions, environmental sampling of air, water, and water-related structures of the hematology unit were conducted; we also performed molecular analysis of patient and environmental isolates. We describe the incidence, clinical presentation, and outcome of these cases and compare these results with cases of superficial *Fusarium* spp. infection among outpatient dermatologic patients at the same hospital.

## Patients and Methods

The University Hospital, Federal University of Rio de Janeiro, Rio de Janeiro, Brazil, is a tertiary-care teaching hospital with ≈450 beds and a hematology unit that has 5 double-bed rooms without high-efficiency particulate air filtration and 8 single-bed rooms equipped with filters and positive pressure. Cases of IF were identified during daily visits to the hematology ward, by review of a database of episodes of febrile neutropenia (active since 1986), and by review of the hospital’s mycology laboratory and pathology registries. Typically, the diagnosis of IF is confirmed by blood culture and/or by direct exam, culture, and histopathology of metastatic skin lesions, when present (*3*).

Cases of superficial infections caused by *Fusarium* spp. were identified by reviewing the Mycology Laboratory database of cultures of dermatologic patients, a database that contains description of the type of lesion from which direct examination and culture were performed, as well as the results of direct exam and culture. These infections were diagnosed in outpatients who attended the hospital’s dermatology clinic, and the diagnosis required the presence of a superficial lesion with positive culture of the lesion.

We reviewed the records of all patients in whom IF was diagnosed during 2000–2010, obtaining detailed information on demographics, underlying disease and treatment, comorbidities, presence of neutropenia, receipt of corticosteroids and other immunosuppressive agents, clinical manifestations of IF, diagnosis, treatment, and outcome. All patients had been hospitalized for the treatment of an underlying hematologic condition and had fusariosis develop in the context of immunosuppression caused by the underlying disease and its treatment. IF was defined as the isolation of *Fusarium* spp. from any sterilized biologic material, such as blood or skin biopsy, or from respiratory secretions in patients with typical clinical signs, including fever and metastatic skin lesions (*1*). A cutaneous portal of entry was defined when the clinical manifestations (and the diagnosis) of IF were preceded by the occurrence of localized skin lesions (such as cellulitis at sites of onychomycosis and intertrigo) with positive culture for *Fusarium* spp. The cases of IF were classified as proven or probable, according to the modified criteria of the European Organization for Research and Treatment of Cancer/Invasive Fungal Infections Cooperative Group and the National Institute of Allergy and Infectious Diseases Mycoses Study Group Consensus Group (*6*). Superficial infections caused by *Fusarium* spp. in immunocompetent patients from the dermatology clinic were defined when *Fusarium* spp. was recovered from a skin lesion (usually onychomycosis and intertrigo). No changes in the population at risk, standards of collection and processing of biologic material, and diagnostic capabilities in the mycology laboratory occurred during the study period.

For the purpose of estimating changes in the incidence of IF, we split the study period into 2 periods: 2000–2005 (period 1) and 2006–2010 (period 2). We calculated the incidence of IF for the 2 periods using total admissions in the hematology unit as denominator and expressing the rates as number of cases per 1,000 admissions. The incidence of superficial infections caused by *Fusarium* spp. was expressed as number of positive cultures per 1,000 superficial cultures processed. Incidence densities between different periods were compared by the χ^2^ test using Epi Info software version 6.04d (Centers for Disease Control and Prevention, Atlanta, GA, USA). We considered p values <0.05 as statistically significant.

## Results

During 2000–2010, a total of 21 cases of IF were diagnosed in patients in the hematology unit at the hospital (Table). Acute myeloid leukemia (AML) was the most frequent underlying disease (42.9%); 12 patients (57.1%) were HCT recipients (8 allogeneic, 4 autologous). Neutropenia (81.0%), receipt of corticosteroids (76.2%), and graft-versus-host disease (6 of 8 allogeneic HCT recipients) were the most frequent predisposing factors. The IF diagnosis was confirmed by blood culture alone in 7 cases, blood culture plus culture and histopathology of biopsy of a metastatic skin lesion in 4, culture and histopathology of skin biopsy in 7, culture of synovial fluid in 2, and culture of sinus aspirate in 1. Among the 20 patients who received treatment, deoxycholate amphotericin B (10 patients) and voriconazole (7 patients) were the most frequent agents used as primary therapy. The overall 30-day and 60-day survival rates for the 21 patients were 33.3% and 28.6%, respectively.

**Table:** Characteristics of 21 patients with invasive fusariosis in the hematology united at University Hospital, Federal University of Rio de Janeiro, Rio de Janeiro, Brazil, 2000–2010*

Characteristic	Patients
Sex, M:F	16:5
Median age, y (range)	55 (9–71)
Underlying disease	
Acute myeloid leukemia	9 (42.9)
Multiple myeloma	4 (19.0)
Non-Hodgkin lymphoma	2 (9.5)
Acute lymphoid leukemia	2 (9.5)
Myelodysplasia	2 (9.5)
Aplastic anemia	1 (4.8)
Chronic myeloid leukemia	1 (4.8)
HCT recipients	12 (57.1)
Allogeneic	8 (38.1)
Autologous	4 (19.0)
Room with HEPA filter	14 (66.7)
Receipt of corticosteroids	16 (76.2)
Graft-versus-host disease, n = 8†	6 (75.0)
Neutropenia	17 (81.0)
Skin as portal of entry	17 (81.0)
Positive blood culture	11 (52.4)
Classification of fusariosis	
Proven	20 (95.2)
Probable	1 (4.8)
Primary treatment	
None	1 (4.8)
Voriconazole	7 (33.3)
Deoxycholate amphotericin B	10 (47.6)
Deoxycholate amphotericin B + voriconazole	3 (14.3)

*Values are no. (%) patients except as indicated. HCT, hematopoietic cell transplant; HEPA, high-efficiency particulate air. †Allogeneic HCT recipients.

**Figure 1:**
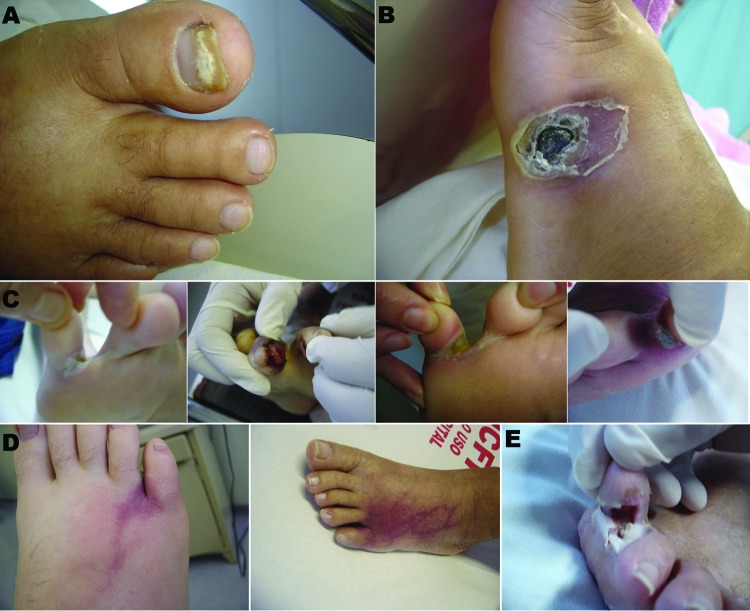
Primary skin lesions in patients with invasive fusariosis in the hematology unit at University Hospital, Federal University of Rio de Janeiro, Rio de Janeiro, Brazil, 2000–2010. A) Onychomycosis; B) ulcer; C) intertrigus; D) intertrigus evolving to lymphangitis before dissemination. (First image in D is the same patient as the first image in C; second image in D is the same patient as the fourth image in C.) E) Necrosis in a lesion of intertrigus (evolution of the lesion shown in the third image in C).

A cutaneous portal of entry was identified in 14 (66.7%) of the 21 cases: onychomycosis with periungueal cellulitis in 6 cases, onychomycosis with interdigital intertrigo in 1 case, intertrigo with lymphangitis in 2 cases, intertrigo alone in 4 cases, and ulcer in 1 case (Figure 1, panels A–D). Some of the lesions of intertrigo evolved to necrosis and tissue loss (Figure 1, panel E). The median time from admission to recognition of a cutaneous portal of entry was 11 days (range −11 to 65).

**Figure 2:**
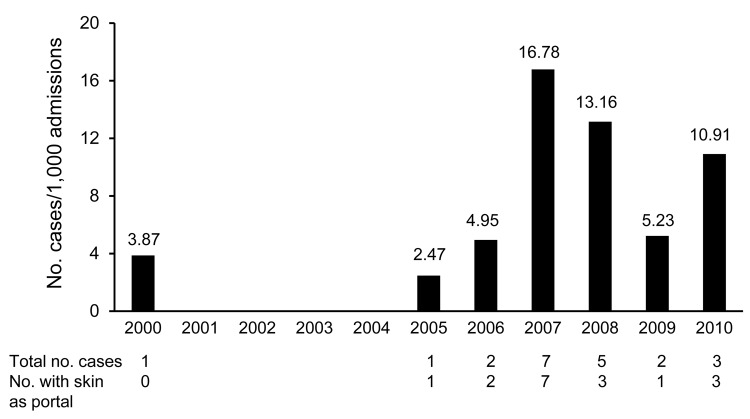
Incidence of invasive fusariosis among patients in the hematology unit at University Hospital, Federal University of Rio de Janeiro, Rio de Janeiro, Brazil, 2000–2010.

Figure 2 shows the incidence of IF during the full study period. The incidence was 0.86 cases per 1,000 admissions in period 1 and 10.23 cases per 1,000 admissions in period 2 (p<0.001). The incidence of IF with a cutaneous portal of entry was 0.43 per 1,000 admissions in period 1 and 6.99 per 1,000 admissions in period 2 (p<0.001).

**Figure 3:**
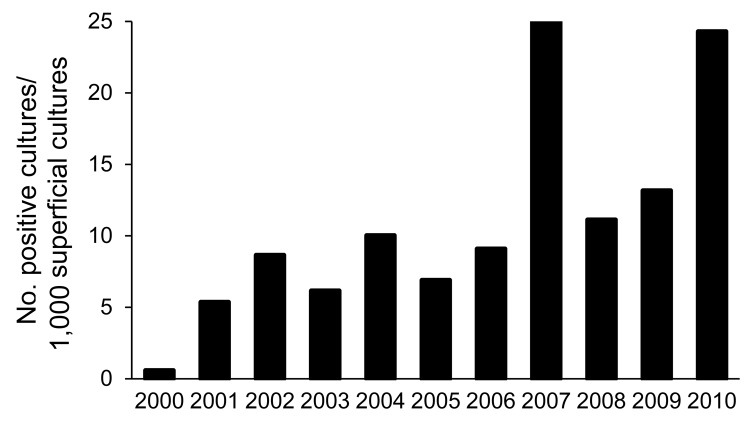
Incidence of superficial infections caused by *Fusarium* spp. among outpatients at the dermatology clinic of University Hospital, Federal University of Rio de Janeiro, Rio de Janeiro, Brazil, 2000–2010.

Figure 3 shows the incidence of superficial infection caused by *Fusarium* spp. in patients from the dermatology outpatient clinic. The incidence (positive cultures per 1,000 superficial cultures) was 7.23 in period 1 and 16.26 in period 2 (p<0.001).

Isolates from hematologic and dermatologic patients showed similar species distribution, with a predominance of *F. solani* species complex (FSSC) species 2 (69% and 74%, respectively). By contrast, most environmental isolates from the initial investigation were identified as *F. oxysporum* species complex, with very few isolates of FSSC species 2, suggesting that most of the clinical cases of IF we identified would not be traceable to a specific environmental source in the hospital ward (*7*).

## Discussion

We observed an increase in the incidence of IF over time among patients in the hematology ward of our hospital; a cutaneous portal of entry was evident in most cases. We also observed an increase in the incidence of superficial infections caused by *Fusarium* spp. in outpatient nonhematologic patients at the same hospital during the same period.

The increase in the incidence of IF from period 1 (2000–2005) to period 2 (2006–2010) was >10-fold and showed a clear upward trend. Although IF is considered an emerging invasive fungal disease, affecting mostly patients with hematologic malignancies (*3*), its incidence is usually low. An epidemiologic study conducted in 18 hospitals in Italy reported 15 cases of IF among 11,802 patients with hematologic malignancies; patients with AML had the highest incidence (13 cases in 3,012 patients) (*8*). Another Italian study reported 3 cases among 1,249 allogeneic HCT recipients (*9*). In the United States, a large prospective study performed in 21 centers (1,194 allogeneic HCT recipients) reported only a few cases of infection caused by non–*Aspergillus* spp. molds, with a <0.3% 1-year cumulative incidence (*10*). By contrast, a prospective study conducted in 8 centers in Brazil during 2007–2009 reported 23 episodes of IF in 937 hematologic patients (2.4% overall incidence rate) (*11*); this study found a 1-year cumulative incidence of 5.2%, 3.8%, and 0.6% among allogeneic HCT recipients, AML patients, and autologous HCT recipients, respectively. No center effect was observed to account for this high incidence.

We also found a high incidence of a cutaneous portal of entry for IF, which is in sharp contrast with what had been previously reported. Similar to invasive aspergillosis, IF is thought to be acquired by inhalation of conidia from the air but occasionally has a cutaneous portal of entry (*4*). In a review of 259 published cases of IF, a cutaneous portal of entry was reported for only 11% of cases, and these were nearly all restricted to onychomycosis as the primary lesion (*5*). By contrast, 14 (66.7%) of the 21 IF cases in our study had a cutaneous portal of entry. Molecular typing of isolates recovered from sites of invasive disease (blood, synovial fluid) and from the lesions in the feet thought to be the portal of entry was performed for 4 cases and showed the same species for 3 (*7*).

Our results showed that interdigital intertrigo was as common as onychomycosis, occurring in 7 of the 14 cases with a cutaneous portal of entry. Onychomycosis is usually caused by *Candida* spp. and dermatophytes (*12*), but fusarial onychomycosis is a known clinical entity (*13*). Furthermore, recent studies have suggested that nondermatophyte fungi (including *Fusarium* spp.) are emerging as causes of onychomycosis (*14*–*16*). By contrast, interdigital intertrigo is rarely caused by *Fusarium* spp. (*17*).

The increased incidence of IF observed in 2007 at our hospital and the unique aspect of a cutaneous portal of entry in most cases raised the possibility that the patients could have acquired IF by contact with contaminated water in the hospital. Our hypothesis was that patients had been admitted with subtle skin breakdowns that became colonized by *Fusarium* spp. after contact with the hospital water, and local infection and dissemination subsequently developed. In support of this hypothesis were the findings of Anaissie et al., who reported that *Fusarium* spp. were recovered from 57% of water samples and 88% of water-related structures in a hospital in the United States; molecular studies of the isolates revealed a close relatedness between patient and environmental isolates (*18*). However, our environmental investigation showed that, although *Fusarium* spp. were present in the hospital water system, most isolates from patients belonged to the FSSC 2, whereas environmental isolates belonged to the *F. oxysporum* species complex (*7*). These results suggest that the infection did not have a nosocomial origin.

Concomitant to the increase in IF, we recorded an increase in the growth of *Fusarium* spp. from superficial infections in outpatients, from 7.23 positive cultures for period 1 to 16.26 positive cultures per 1,000 superficial cultures for period 2. Considering this apparent emergence of fusarial superficial infections in the community, the immunocompromised patients served as sentinels for the detection of this problem (*19*).

A limitation of our study is the denominator used to calculate the incidence. Because infection was acquired in the community, the appropriate denominator would be population based. However, because obtaining such a denominator would be difficult, we used a hospital-based denominator to approximate the incidence.

Our findings may have implications for future research, in particular, determining the environmental reservoirs of *Fusarium* spp. in the community that promoted the emergence of superficial fusariosis in immunocompetent patients. *Fusarium* spp. are widely found in the environment and are pathogens of various plants, including tomatoes, soybeans, and various grains (*20*). One possibility for an increase in *Fusarium* spp. in the environment is agricultural activities. For example, the Cerrado area is a large (≈2 million m^2^) territory that encompasses 10 states of Brazil. During the past 15–20 years, the area underwent a great deal of change in its composition, with a massive replacement of the native vegetation with monoculture, typically soybeans and pasture (*21*). A study evaluating the fungal diversity of the region found a great loss of fungal richness and diversity in the soybean plantation and pasture areas compared with native vegetation, with a concentration of ascomycetes (*22*). Other questions that require future research include assessment of the frequency and clinical significance of baseline skin colonization with *Fusarium* spp. in immunosuppressed patients and evaluation of preventive measures to reduce the incidence of this devastating disease.

In conclusion, we observed an increase in the incidence of IF in our hematology ward, with a cutaneous portal of entry, and of superficial fusariosis in immunocompetent outpatients. Future studies are needed to identify reservoirs of *Fusarium* spp. in the community, as well as preventive measures for patients at high risk for IF.

## References

[1] Nucci M, Anaissie EJ, Queiroz-Telles F, Martins CA, Trabasso P, Solza C, Outcome predictors of 84 patients with hematologic malignancies and *Fusarium* infection. Cancer. 2003;98:315–9. 10.1002/cncr.11510 12872351

[2] Nucci M, Marr KA, Queiroz-Telles F, Martins CA, Trabasso P, Costa S, *Fusarium* infection in hematopoietic stem cell transplant recipients. Clin Infect Dis. 2004;38:1237–42 and. 10.1086/383319 15127334

[3] Nucci M, Anaissie E. Emerging fungi. Infect Dis Clin North Am. 2006;20:563–79 and. 10.1016/j.idc.2006.06.002 16984869

[4] Girmenia C, Arcese W, Micozzi A, Martino P, Bianco P, Morace G. Onychomycosis as a possible origin of disseminated *Fusarium solani* infection in a patient with severe aplastic anemia. Clin Infect Dis. 1992;14:1167 and. 10.1093/clinids/14.5.1167 1534694

[5] Nucci M, Anaissie E. Cutaneous infection by *Fusarium* species in healthy and immunocompromised hosts: implications for diagnosis and management. Clin Infect Dis. 2002;35:909–20 and. 10.1086/342328 12355377

[6] De Pauw B, Walsh TJ, Donnelly JP, Stevens DA, Edwards JE, Calandra T, Revised definitions of invasive fungal disease from the European Organization for Research and Treatment of Cancer/Invasive Fungal Infections Cooperative Group and the National Institute of Allergy and Infectious Diseases Mycoses Study Group (EORTC/MSG) Consensus Group. Clin Infect Dis. 2008;46:1813–21 and. 10.1086/588660 18462102 PMC2671227

[7] Scheel CM, Hurst SF, Barreiros G, Akiti T, Nucci M, Balajee SA. Molecular analyses of *Fusarium* isolates recovered from a cluster of invasive mold infections in a Brazilian hospital. BMC Infect Dis. 2013;13:49 and. 10.1186/1471-2334-13-49 23363475 PMC3579725

[8] Pagano L, Caira M, Candoni A, Offidani M, Fianchi L, Martino B, The epidemiology of fungal infections in patients with hematologic malignancies: the SEIFEM-2004 study. Haematologica. 2006;91:1068–75 . 16885047

[9] Pagano L, Caira M, Nosari A, Van Lint MT, Candoni A, Offidani M, Fungal infections in recipients of hematopoietic stem cell transplants: results of the SEIFEM B-2004 study—Sorveglianza Epidemiologica Infezioni Fungine Nelle Emopatie Maligne. Clin Infect Dis. 2007;45:1161–70 and. 10.1086/522189 17918077

[10] Kontoyiannis DP, Marr KA, Park BJ, Alexander BD, Anaissie EJ, Walsh TJ, Prospective surveillance for invasive fungal infections in hematopoietic stem cell transplant recipients, 2001–2006: overview of the Transplant-Associated Infection Surveillance Network (TRANSNET) Database. Clin Infect Dis. 2010;50:1091–100 and. 10.1086/651263 20218877

[11] Nucci M, Garnica M, Gloria AB, Lehugeur DS, Dias VC, Palma LC, Invasive fungal diseases in haematopoietic cell transplant recipients and in patients with acute myeloid leukaemia or myelodysplasia in Brazil. Clin Microbiol Infect. 2012. [Epub ahead of print]. 10.1111/1469-0691.12002 23009319

[12] Thomas J, Jacobson GA, Narkowicz CK, Peterson GM, Burnet H, Sharpe C. Toenail onychomycosis: an important global disease burden. J Clin Pharm Ther. 2010;35:497–519 and. 10.1111/j.1365-2710.2009.01107.x 20831675

[13] Nucci M, Anaissie E. *Fusarium* infections in immunocompromised patients. Clin Microbiol Rev. 2007;20:695–704 and. 10.1128/CMR.00014-07 17934079 PMC2176050

[14] Gupta AK, Ryder JE, Baran R, Summerbell RC. Non-dermatophyte onychomycosis. Dermatol Clin. 2003;21:257–68 and. 10.1016/S0733-8635(02)00086-4 12757248

[15] Guilhermetti E, Takahachi G, Shinobu CS, Svidzinski TI. *Fusarium* spp. as agents of onychomycosis in immunocompetent hosts. Int J Dermatol. 2007;46:822–6 and. 10.1111/j.1365-4632.2007.03120.x 17651164

[16] Godoy-Martinez P, Nunes FG, Tomimori-Yamashita J, Urrutia M, Zaror L, Silva V, Onychomycosis in Sao Paulo, Brazil. Mycopathologia. 2009;168:111–6 and. 10.1007/s11046-009-9209-5 19424818

[17] Calado NB, Sousa F Jr, Gomes NO, Cardoso FR, Zaror LC, Milan EP. *Fusarium* nail and skin infection: a report of eight cases from Natal, Brazil. Mycopathologia. 2006;161:27–31 and. 10.1007/s11046-005-0136-9 16389481

[18] Anaissie EJ, Kuchar RT, Rex JH, Francesconi A, Kasai M, Muller FM, Fusariosis associated with pathogenic *Fusarium* species colonization of a hospital water system: a new paradigm for the epidemiology of opportunistic mold infections. Clin Infect Dis. 2001;33:1871–8 and. 10.1086/324501 11692299

[19] Rubin RH. The compromised host as sentinel chicken. N Engl J Med. 1987;317:1151–3 and. 10.1056/NEJM198710293171809 3657881

[20] Osborne LE, Stein JM. Epidemiology of *Fusarium* head blight on small-grain cereals. Int J Food Microbiol. 2007;119:103–8 and. 10.1016/j.ijfoodmicro.2007.07.032 17716761

[21] Brannstrom C, Jepson W, Filippi AM, Redo D, Xu Z, Ganesh S. Land change in the Brazilian Savanna (Cerrado), 1986−2002: comparative analysis and implications for land-use policy. Land Use Policy. 2008;25:579–95. 10.1016/j.landusepol.2007.11.008

[22] de Castro AP, Quirino BF, Pappas G Jr, Kurokawa AS, Neto EL, Kruger RH. Diversity of soil fungal communities of Cerrado and its closely surrounding agriculture fields. Arch Microbiol. 2008;190:129–39 and. 10.1007/s00203-008-0374-6 18458875

